# Wounds

**Published:** 1861-09

**Authors:** 


### Wounds.

Dr. Mitchell Henry, Surgeon to the Middlesex Hospital, observes that strapping and adhesive materials, except sometimes as tending to support and consolidate the sound textures, should be avoided—metalic sutures, either of well annealed iron wire orof silver wire, being substituted for them. Metallic sutures excite no irritation, and may be, fearlessly employed in every situation and to all textures. Unlike sutures made of vegetable materials, the holes through which they pass undergo no suppuration or ulceration, and they may be left *in situ* for an indefinite period—for three weeks if it be desired; indeed, it is not an uncommon event to lose a wire suture altogether, the patient leaving the hospital with it still in his body. The arnica lotion should be applied over the wounded part, which, when practicable, should also be kept elevated high above the patient's body; and the relief that these two measures afford will suprise persons who are not familiar with them. The only exception to the propriety of elevating the limb is in cases in which there is much loss of temperature from defective circulation, or fear of slouahing from the severity of the injury. To limbs in this condition, thick layers of cotton wool form the most advisable application; but no worse practice can be adopted than that which is too common—namely, the placing of the injured part in a hot poultice that it may "recover itself." The result of careful adjustment, and of attempts to save textures and parts, almost against hope, will amply repay the surgeon, who, at the same time, places his reliance in opium and wine as constitutional treatment. When reaction and inflammatory fever set in, the tincture of aconite, when carefully watched, will exhibit the surprising power which it exerts over all forms of vascular excitement.

*Suppurating sores and wounds.*—Suppuration is a great evil, and may become a great peril to the patient. All applications that promote it are, therefore, to be avoided—such as hot fomentations of all kinds, poultices and ointments. Torn and disorganized parts will suppurate in spite of all that we can do. Our efforts must, then, be directed to rendering the process as little inconvenient as possible. The most scrupulous attention must be paid to supporting the suppurating textures by means of splints, strapping the sound parts of the limb, and applying graduated pressure, so as to limit the area of suppuration, and prevent the matter from burrowing. Next, the pus itself must be rendered as innocuous as possible by attention to cleanliness, and by disinfection when, as is most commonly the case, this also is required. Long ago impressed with the fact that erysipelas, diarrhœa, phlebetis, and low fever, could often be traced to the foul condition of suppurating sores on the patient's own body, Dr. H. tried almost every disinfectant, including of late the mixture of plaster-of-Paris and coal-tar, so much eulogized in France. One objection to it, and to several others—as, for instanee, charcoal, one of the best of all, is that they are unseemly, or smell disagreeably. No such objection, however, applies to a solution of Condy's fluid (the permanganate of potash,) which, for two years past, he has largely and most beneficially employed in the case of all varieties of suppurating sores. A solution of from half a drachm to two or four drachms of the purple fluid, to the pint of water, will keep even the most offensive sore sweet and clean, and, as a general rule, produce no pain whatever. If it does, the solution must be made weaker, until the required strength is attained, for there is a great difference in respect to its tolerance amongst patients. Its benefits are, perhaps, more strikingly displayed in ths case of burns than in any other form of suppurating sore. From the day that suppuration commences in the case of a burn or scald, under his care, it is dressed with lint saturated with a weak solution of this fluid, commencing with half a drachm to the pint of water, over which a layer of cotton wool is applied. The smell, always one of the most distressing features in these cases, is very greatly lessened, if not altogether destroyed; and the patient, no longer tormented with a horrible odor constantly under his nose, regains his appetite for food, whilst he is spared the chances of constitutional affections from absorption of putrid matters.

The worst case of burn Dr. H. ever saw recover, extending, as it did, over nearly all the left side of the trunk and abdomen, by far the most perilous situations, as well as over left upper and lower extremities, was treated with this fluid. In the case of an old woman, from whom was removed a large polypus which had grown into the frontal sinus and displaced the eye, the serious typhoid condition into which she was thrown, by the putrid discharge flowing back into the pharynx, was chiefly relieved by the assiduity with which the discharge was disinfected by injecting the two-drachm solution of Condy's fluid into the nares every four hours. So also it has been employed as a wash for the mouth, as an application to destroy the fetor of cancerous sores, and as an injection in uterine carcinoma. Chemical science teaches us that the permanganates are true disinfectants because they oxygenate, and do not act merely as deodorizers.—*Braithwaites Retrospect, No.* 41—*Summary Med. Science.*

---

*Wounds, (Treatment of) after Operations, &c.*—The following is the very sensible practice of Dr. Humphrey, surgeon to Addenbroke's Hospital, Cambridge.

"*Leaving the wound uncovered.*—It is well known that wounds of the face generally heal up, in their whole length, by first intention. This is due, in great measure, to the vital qualities of the parts; and in some degree also, I apprehend, to the fact that they are usually left exposed to the air, their edges being held in contact merely by sutures. For some years we have adopted this plan after amputations and all, or nearly all, other operations. The integuments are united by sutures placed at intervals of about an inch; and the wound, as well as the adjacent surface, is left quite exposed to the air; no plaster, bandage, or dressing of any kind, being placed upon it. The advantages of this mode of treatment are very great. All the irritation, the galling pressure, the retention of heat, and other inconveniences resulting from bandages and plasters are thus avoided. The edge of the wound and the surrounding skin being uncovered, the eye can take cognizance of what is going on; and we can cut a stitch here and there, when required, can keep the part clean, or take other measures without difficulty. Forasmuch as no dressings are applied, there are none to be removed. The suffering which used to be caused by the dressing of wounds after operations is got rid of. In many cases, I do not touch the wound, except for the purpose of removing the sutures, from the day of the operation; and several patients, who have undergone amputation under chloroform, have told me that, neither during the operation nor subsequently, did they experience any pain whatever. We decidedly have more frequent union by first intention than when we were in the habit of applying dressings to the wound. This not unfrequently takes place throughout the entire wound, except in the one or two narrow tracks where the ligatures pass, after amputation, and even sometimes after excision of joints.

"We close the wound at the time of the operation, while the patient is still under the influence of chloroform. At one time, we tried the plan of leaving the wound open for some hours after the operation; but we did not find any special advantage in it, certainly not enough to compensate the patient for the suffering and disappointment caused by our reappearing to close and stitch up the wound after he was in bed, when he was just beginning to recover from the effects of the operation, and to congratulate himself that he had escaped from our torturings.

"If suppuration takes place, an early and free vent should be afforded to the pus, by cutting the stitches and opening the wound; and care should be taken to keep the wound clean. In this, a syringe is a very useful adjunct, which might be more frequently employed by English surgeons than it is. In Berlin, a syringe is always carried by the surgeon in his visits round the hospital, and is used by him to cleanse almost every wound and sore.

"Water dressing or poultices are sometimes required; and their influence, at times soothing, at others slightly stimulating, is often very beneficial. Commonly, I think, they are used too indiscriminately, and continued too long. They have a tendency to relax the parts, and appear to me to predispose, in some measure, to erysipelas. The quantity of poultice which is used in some hospitals is certainly remarkable, and might, I think, with great advantage, be diminished.

"Large open wounds—that is where portions of the skin have been removed, so that the edges cannot be approximated—are, in our hospital, not unfrequently, left exposed to the air, without any covering. A dry crust or scab forms upon them, beneath which cicatrization goes on; and we find that the healing often proceeds more quickly in this way than when the part is kept moist and the products of the wound are continually flowing away into the poultices or dressings.

"*Adhesive plaster applied wet.*—When it is desirable to use adhesive plaster, you will find it an excellent plan to steep the pieces of plaster in hot water before applying it, and to put it upon the part while it is moist. In this state, it can be adapted to the service more accurately than when it is heated by the fire and applied in the usual manner, and it is decidedly less irritating. It soon dries, and, when dry, adheres well. This method is very convenient in the case of slight wounds of the hand; for when the plaster has become dry, it forms a very effective protection. It often remains on for days, in spite of repeated washing of the hands. We find the wetted plaster of great service in the treatment of sore legs, swelled joints, and other cases where pressure is required, as well as in the management of wounds.—*Brit. Med. Jour.*, *Oct.*, 1860.—*Summary Med. Science.*

